# Screens in fly and beetle reveal vastly divergent gene sets required for developmental processes

**DOI:** 10.1186/s12915-022-01231-4

**Published:** 2022-02-08

**Authors:** Muhammad Salim Hakeemi, Salim Ansari, Matthias Teuscher, Matthias Weißkopf, Daniela Großmann, Tobias Kessel, Jürgen Dönitz, Janna Siemanowski, Xuebin Wan, Dorothea Schultheis, Manfred Frasch, Siegfried Roth, Michael Schoppmeier, Martin Klingler, Gregor Bucher

**Affiliations:** 1 Johann-Friedrich-Blumenbach-Institut, GZMB, University of Göttingen, Justus-von-Liebig-Weg 11, 37077 Göttingen, Germany; 2 Current address: Institute of Clinical Pharmacology, University Medical Center Göttingen, University of Göttingen, Robert-Koch-Str. 40, 37075 Göttingen, Germany; 3 Department of Biology, Friedrich-Alexander-University Erlangen-Nürnberg, Staudtstr. 5, 91058 Erlangen, Germany; 4 Current address: Department of Medical Bioinformatics, University Medical Center Göttingen, University of Göttingen, Goldschmidtstr. 1, 37077 Göttingen, Germany; 5 Current address: Department of Insect Biotechnology, Justus-Liebig-University Giessen, Heinrich-Buff-Ring 26-32, 35392 Gießen, Germany; 6 Current address: Institute of Pathology, University Hospital Cologne, Kerpener Str. 62, 50924 Cologne, Germany; 7 Current address: Institute of Neuropathology, University Hospital Erlangen, Friedrich-Alexander-University Erlangen-Nürnberg, Erlangen, Germany; 8 Institute for Zoology/Developmental Biology, University of Cologne, Biocenter, Zülpicher Straße 47b, D-50674 Köln, Germany

**Keywords:** Gene function, Comparative genomics, RNAi screen, iBeetle, *Tribolium castaneum*, *Drosophila melanogaster*, Divergence of gene function, iBeetle-Base, FlyBase

## Abstract

**Background:**

Most of the known genes required for developmental processes have been identified by genetic screens in a few well-studied model organisms, which have been considered representative of related species, and informative—to some degree—for human biology. The fruit fly *Drosophila* melanogaster is a prime model for insect genetics, and while conservation of many gene functions has been observed among bilaterian animals, a plethora of data show evolutionary divergence of gene function among more closely-related groups, such as within the insects. A quantification of conservation versus divergence of gene functions has been missing, without which it is unclear how representative data from model systems actually are.

**Results:**

Here, we systematically compare the gene sets required for a number of homologous but divergent developmental processes between fly and beetle in order to quantify the difference of the gene sets. To that end, we expanded our RNAi screen in the red flour beetle *Tribolium castaneum* to cover more than half of the protein-coding genes. Then we compared the gene sets required for four different developmental processes between beetle and fly. We found that around 50% of the gene functions were identified in the screens of both species while for the rest, phenotypes were revealed only in fly (~ 10%) or beetle (~ 40%) reflecting both technical and biological differences. Accordingly, we were able to annotate novel developmental GO terms for 96 genes studied in this work. With this work, we publish the final dataset for the pupal injection screen of the *iBeetle screen* reaching a coverage of 87% (13,020 genes).

**Conclusions:**

We conclude that the gene sets required for a homologous process diverge more than widely believed. Hence, the insights gained in flies may be less representative for insects or protostomes than previously thought, and work in complementary model systems is required to gain a comprehensive picture. The RNAi screening resources developed in this project, the expanding transgenic toolkit, and our large-scale functional data make *T. castaneum* an excellent model system in that endeavor.

**Supplementary Information:**

The online version contains supplementary material available at 10.1186/s12915-022-01231-4.

## Background

The function of genes is paramount for the biology of any organism and, hence, the assignment of functions to genes is a central question of biological research. However, only in a very small number of genetic model species like the mouse *Mus musculus*, the zebrafish *Danio rerio*, the nematode *Caenorhabditis elegans,* and the vinegar fly *Drosophila melanogaster* have the functions of developmental genes been assayed in systematic screens. This restriction to a few model systems is a consequence of the necessity for an elaborate genetic and molecular tool kit, which is extremely laborious to establish [1–4]. Unfortunately, it has remained unclear how representative findings in these model species actually are for their clade or in other words, how quickly and profoundly gene function diverges in evolution. Indeed, based on an astonishing degree of functional conservation with respect to many genes, it has been assumed that the findings gained in those few model species can be transferred to a large degree. This assumption underlies the use of the insect *D. melanogaster* as a model for a number of human diseases, including developmental aspects like heart formation. Conversely, even among insects, dramatic evolutionary changes of gene functions have been described with respect to homologous developmental processes, calling into question the degree of transferability. However, a systematic comparison of the gene sets required for the same process in different species has not been done and, hence, a quantification of divergence versus conservation remains badly missing. Importantly, knowing the degrees of gene function divergence is relevant not only for understanding the evolution of biodiversity but also for applied research, e.g., for transferring knowledge from model systems to species relevant for medical applications or pest control.

Recently, the study of gene function in development has been extended to non-traditional model organisms. Predominantly, candidate genes known for their function in the classical model systems have been tested in other organisms. Subsequent comparisons revealed both, conservation and divergence of gene functions. For example, axis formation in *D. melanogaster* has turned out to be a rather diverged process partially based on different genes compared to other insects. The key anterior morphogen of *D. melanogaster*, *bicoid*, is not present in most insects [5]. Instead, repression of Wnt signaling plays a central role in the red flour beetle *Tribolium castaneum* [6] as it does in many animals including other insects, flatworms, and vertebrates [7–10] - but not in *D. melanogaster*. The functions of genes of the Hox cluster, in contrast, appear conserved over very large phylogenetic distances—although some functional divergence has been linked to the evolution of arthropod morphology [11]. Likewise, the gene regulatory network of dorso-ventral patterning and head specification show the involvement of similar gene sets, although a few components appear to be required for only some clades [12–15].

Notably, the differences in gene functions documented so far may be an underestimation of the real divergence, because the prevailing candidate gene approach leads to a systematic bias towards conservation. The genes to be tested are usually chosen based on the knowledge of their ortholog’s involvement in other species. As a consequence, unrelated genes are rarely tested and the involvement of unexpected genes in a given process is underestimated. Hence, approaches are needed to overcome this bias and to gain a realistic view on the degree of gene function divergence. To that end, genes required for certain biological processes need to be identified in an unbiased and genome-wide manner in non-traditional organisms as well, even though this has remained technically challenging and such large datasets outside the classic model systems had not been available.

The red flour beetle *T. castaneum* has recently been established as the only arthropod model organism apart from *D. melanogaster* where genome-wide unbiased RNAi screens are feasible. Based on the robust and systemic RNAi response of this species, the *iBeetle* large-scale screen was performed where random genes were knocked down and the resulting animals were scored for a number of developmental phenotypes [16–18]. However, the data gained in the *iBeetle* screen had covered only one third of the gene set, not allowing for robust genome wide statements. Apart from its particularly strong and robust RNAi response, *T. castaneum* offers a comparably large tool kit for analyzing gene function including transgenic and genome editing approaches [19–21].

In this paper, we used an expanded dataset to assess the degree of divergence of the gene sets required for selected developmental processes between fly and beetle such as head, muscle and ovary development, and dorso-ventral patterning. These processes are homologous but show a different degree of evolutionary divergence, which could be reflected in changes of gene function. First, we determined genes that were essential in the beetle for these processes but which had so far not been connected to them in *D. melanogaster*. These a priori unexpected genes sum up to about 37% of the total genes identified to be required for either one or both species. For 30% of these genes, no functional annotation had been available at FlyBase at all such that we provide the first functional Gene Ontology (GO) assignment for the respective ortholog group in insects. Only two genes essential in *T. castaneum* did not have an ortholog in *D. melanogaster*, i.e., these processes seem not much affected by gene gain or loss. We conclude that restricting genetic screens to one model system only falls short of identifying a comprehensive set of essential genes. Further, our data reveals an unexpected degree of divergence of gene function between two holometabolous insect species. Moreover, we present here an update of the dataset gained in the genome wide *iBeetle* screen in *T. castaneum*. Our analysis is based on both, a dataset previously published comprising 5300 genes [17] and an additional 3200 genes screened as part of this project. In addition to those, we publish and make accessible (at *iBeetle-Base*) the phenotypes for an additional 4520 genes which were screened while the analysis presented here was ongoing. Hence, with this paper, the coverage of genes tested and annotated at iBeetle-Base sums to 13,020 Tribolium genes (78% of the predicted gene set), which will be the final number for the pupal injection screen of the *iBeetle *screen.

## Results

### Reaching the final dataset for pupal injections of the large scale *iBeetle* screen

**Fig. 1:**
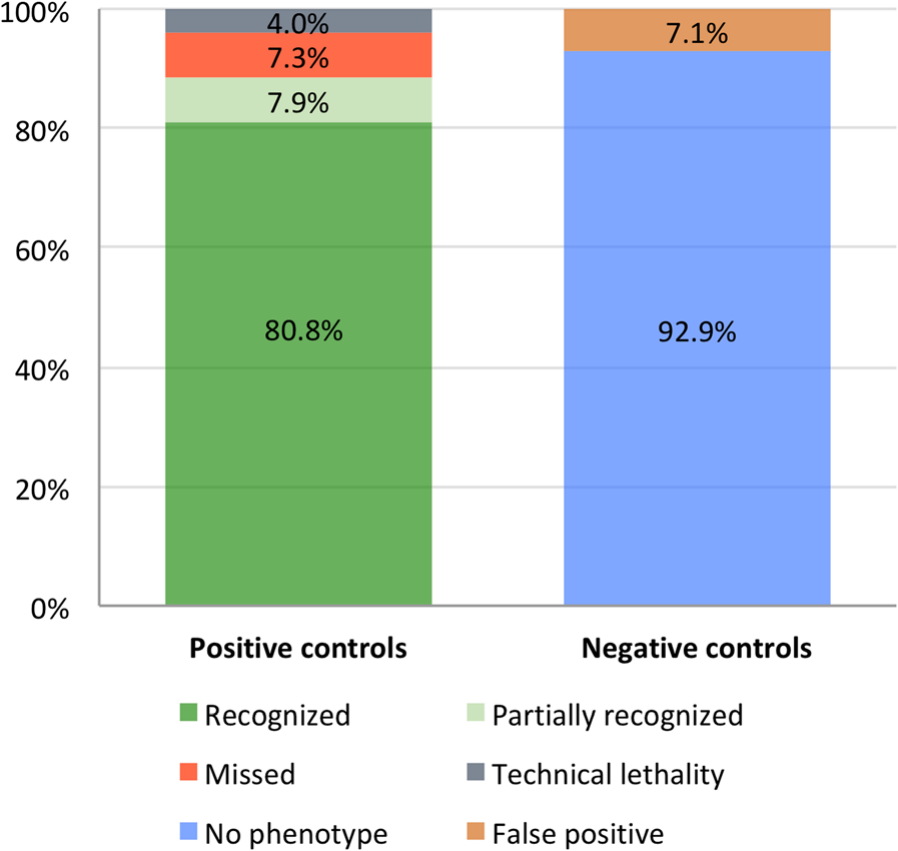
Quality controls of the primary screen. 178 positive controls using 35 different genes were included. More than 88% of the positive controls were fully or partially recognized (left bar) while 7.3% were missed. 4% could not be analyzed due to technical lethality before the production of offspring. 7.1% of the negative controls had annotations, i.e. they were false positive (right bar). These figures are similar to the first screening phase [17]

We added 3200 genes to the previously published 5300 genes of our large-scale *iBeetle* screen [17], reaching a coverage of 51% of the *T. castaneum* gene set of a total of 16,593 currently annotated genes [22]. We followed the previously described procedure for the pupal injection screen [17] with minor modifications (see the “Methods” section). In short, we injected 10 female pupae per gene with dsRNAs (concentration 1 μg/μl). We annotated the phenotypes of the injected animals and the first instar cuticle of their offspring using the EQM system [23], the *T. castaneum* morphological ontology *Tron* [24], and a controlled vocabulary. The data is available at the online database *iBeetle-Base* [25–27]. As positive controls, an array of genes with known phenotypes related to the processes under scrutiny was added to the screening and included both drastic and mild phenotypes (find details in the extensive description of the first part of the screen in Schmitt-Engel et al. [17]). Buffer injections were performed as negative controls. These controls revealed a similar portion of false negative and false positive data compared to the first part of the screen (Fig. 1 and Additional file 1: Table S1). The analysis presented in this work is based on all genes that had been screened in the first round of the screen and the set of genes published with this publication. Taken together, the analyzed set of genes covered approximately 50% of the genome. About two thirds of the *Drosophila* genes relevant for our analysis had been covered by the screen (Additional file 2: Fig. S1). While this analysis was ongoing, we continued the screen as well and have in the meanwhile reached a coverage of 78% (13,020 genes). We publish these additional phenotypic data (accessible online at *iBeetle-Base)* with this article*,* but they were not included in the detailed analysis presented here because both analyses ran in parallel.

### Unexpected gene functions in developmental processes

We wanted to use our large-scale phenotypic dataset to systematically compare the gene sets required for the same biological processes in *T. castaneum* and *D. melanogaster*. We define a gene to be “required for” a process, if its knock-down or mutation leads to a phenotype in that process. To that end, we first identified in an unbiased way all genes annotated with a phenotype for a number of biological processes by searching *iBeetle-Base*. Specifically, we scored for phenotypes indicative of functions in dorso-ventral patterning, head and muscle development, and in oogenesis.

The choice of the processes to be compared was based on two arguments. On one hand, we chose processes that fell within our core expertise for sake of correct interpretation of phenotypes, for the opportunity for subsequent detailed work, and to ensure comprehensive knowledge of respective *Drosophila* data. On the other hand, we aimed at including developmental processes with different degrees of conservation. We assumed that cellular processes such as muscle development might be conserved. Indeed, for both, conservation of basic mechanisms between *Drosophila* and vertebrates was shown along with evolutionary differences [28–31], and the use of *Drosophila* as a model for vertebrate heart development is widely adopted [32, 33]. Some aspects of dorso-ventral patterning are highly conserved among animals (e.g., the involvement of dpp/BMP versus sog) but differences even between insects have been described as well [14, 34]. Likewise, a set of highly conserved genes is involved in anterior development in all animal clades while clear differences were described even between flies and beetles [12, 35–39]. This divergence may to some degree be due to the fly-specific involution and reduction of the larval head. Finally, telotrophic oogenesis of *Tribolium* is morphologically quite distinct from the polytrophic oogenesis found in *Drosophila* [40–42] and both are quite different from the vertebrate process probably representing a more divergent process.

For all these processes, we found a number of gene functions that were expected based on *D. melanogaster* knowledge (see below). This confirmed that the screen design allowed the detection of these types of phenotypes. Importantly, we also found functions for genes so far not connected to those processes (based on FlyBase information [43, 44], PubMed searches, and scientist expertise). The *iBeetle* screen is a first pass screen with a focus on minimizing false negative results with the trade-off of allowing for false positive annotations [17]. The likelihood for this type of error is further increased by off-target effects and/or by strain-specific differences in the phenotype, i.e. genes for which the described phenotype was not reproduced in another genetic background, which we counted as “false positive” in order to be conservative [45]. Hence, we aimed at excluding false positive annotations for the unexpected gene functions. First, we based our analyses only on genes for which phenotypes had been annotated with a penetrance of > 50% in the primary screen. Further, we only used phenotypes that were reproduced by RNAi experiments with non-overlapping dsRNA fragments targeting the same gene. In order to exclude genetic background effects, we used another lab strain (our standard lab strain *San Bernardino*, *SB*) except for the muscle project where we needed to use the *pBA19* strain, which has EGFP marked muscles [46]. This re-screening procedure resulted in a set of genes for which we can claim with high confidence that they are indeed required for these processes in *T. castaneum*, but which previously were not assigned to these in *D. melanogaster* (Additional file 3: Table S2).

### Assigning the first function to a gene versus extending previous annotations

**Fig. 2:**
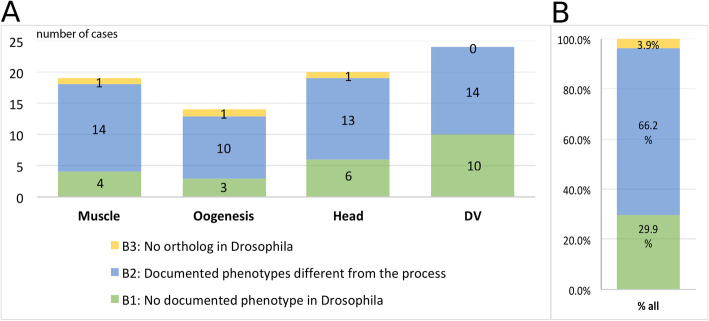
Analysis of genes with unexpected gene functions found in *Tribolium*. **A** Numbers of genes, for which an unexpected function in the respective process was found in the iBeetle screen but had not been known from *Drosophila*. **B** Combined numbers for all four processes. Only three genes with novel gene functions in *Tribolium* had no ortholog in *Drosophila* (yellow). About two-thirds of genes with novel function had previous phenotypic annotations in FlyBase but relating to other biological processes (blue). Importantly, for one third of those genes, we had detected the first phenotype in any insect (green). We added this novel information to the GO database

One reason for a lack of respective functional data in FlyBase could be that the knocked-down beetle gene does not have an ortholog in the fly. In order to test this, we searched for the fly orthologs in orthoDB and by manually generating phylogenetic trees based on searching *T. castaneum*, *D. melanogaster*, and *M. musculus* genomes for orthologs and paralogs. This analysis revealed that only three genes with a novel function (appr. 3%) did not have a *D. melanogaster* ortholog (yellow in Fig. 2; see Additional file 2: Fig. S2 and S3 for phylogenetic trees). Evidently, lineage-specific gene loss or gain explains only a minor part of the functional divergence of homologous developmental processes.

Next, we asked whether the respective *D. melanogaster* orthologs were known to be involved in other biological processes or lacked any phenotype information. For this analysis, we did not only use the genes identified in the screen but included those that had previously been studied, as well. We looked up phenotype information of the respective *D. melanogaster* orthologs on FlyBase (homology assignment done with OrthoDB v9). Among the fly orthologs whose functional annotations did not match with those from the iBeetle screen or published records of functional data, around two thirds (64.6%) had annotations that were related to other processes than the ones studied in *T. castaneum* (Fig. 2). Importantly, one third of the genes (32.3%) did not have any functional annotation in FlyBase. Hence, for those genes, the *iBeetle*-screen had detected the first documented function of that ortholog group in insects. Importantly, due to the lack of previous phenotypic information, these genes likely would not have been included in a classical candidate gene approach.

### A quarter of *Drosophila* gene function annotations were not confirmed for *T. castaneum*

**Fig. 3:**
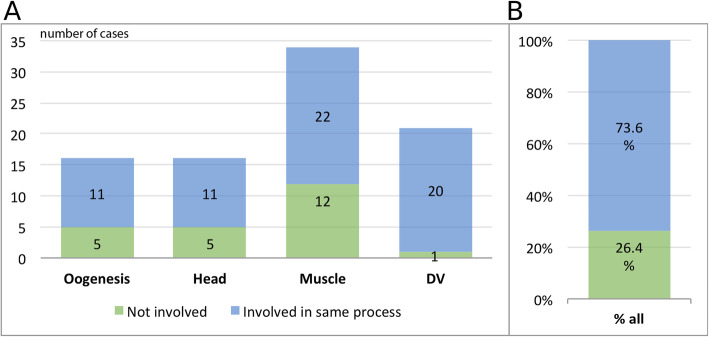
Beetle genes showing phenotypes expected from *Drosophila.* **A** Gene sets known to be required for a given process in *Drosophila* were compared to iBeetle data. Close to three quarters showed related phenotypes (blue) while others had no or different types of phenotypes (green). **B** Approximately one quarter of the genes known to be required for certain *Drosophila* processes were not required for that process in *Tribolium*. This analysis is based on the subset of genes which already had been screened in *Tribolium* (51%). Interestingly, we found orthologs for 66% of the respective *Drosophila* genes—this indicates that our screen was enriched for relevant genes

In a complementary approach, we asked how many genes known to be required for a given process in *D. melanogaster* had been assigned related functions in the *iBeetle* screen. To that end, we first collected lists of genes required for those processes based on *D. melanogaster* knowledge (expert knowledge, literature, and FlyBase) (Additional file 4: Table S3). Then we mined *iBeetle-Base* to see how many of the beetle orthologs had an annotation related to that process (Fig. 3A). About two-thirds of those genes had actually been screened in *T. castaneum* (Additional file 2: Fig. S1) and all following numbers are based on the analysis of this subset.

A surprisingly large portion of genes (26.4%) known to be required for these processes in *D. melanogaster* did not show the expected phenotype in *T. castaneum* (Fig. 3B).

### Enriching the GO information with data from *Tribolium*

Gene ontology (GO) assignment is a powerful tool to establish hypotheses on the function of given gene sets [47]. So far, there were no GO terms associated based on *T. castaneum* data. The work presented here revealed that a surprisingly high portion of orthologous genes has diverging functions in different organisms. To enrich the GO database, we submitted GO terms with respect to the biological process for all 96 re-screened genes with functions in dorso-ventral patterning (GO:0010084), oogenesis (GO:0048477), the development of embryonic muscles (GO:0060538), and head (GO:0048568) [48].

## Discussion

### Investigating one species falls short of a comprehensive view on gene function

Large-scale screens in the leading insect model organism *D. melanogaster* have revealed gene sets required for certain biological processes. As consequence, insect-related GO term annotations are almost exclusively based on work in flies. However, there are several reasons to believe that the picture has remained incomplete. On one hand, species-specific or technical limitations may have prohibited the identification of an involved gene in *D. melanogaster*. On the other hand, evolution has led to functional changes such as the modification or loss of ancestral gene functions or the co-option of genes into a novel process. Unfortunately, it has remained unclear to what extent the gene sets determined exclusively in flies would be representative of insects as a whole or if it is even appropriate to assume the existence of representative gene sets.

**Fig. 4:**
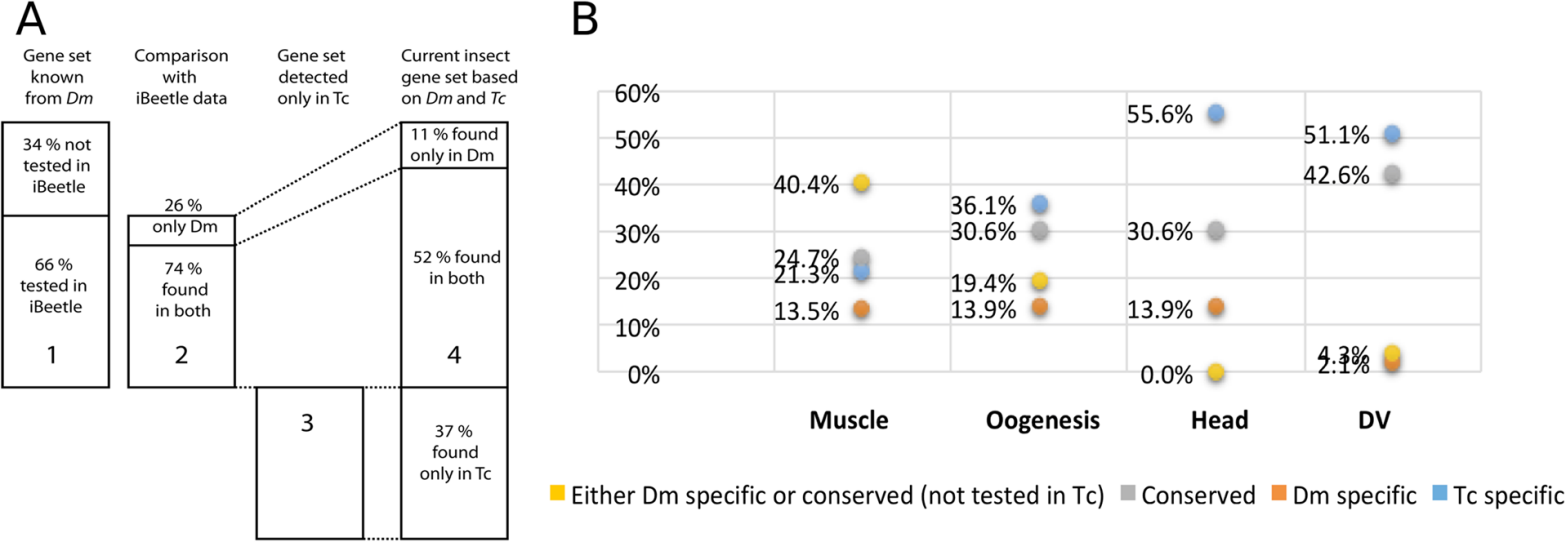
Many genes involved in a given process are detected only in one of the two species. We combined all genes found in the fly to be involved in our processes (column 1) and/or those genes that we identified in the iBeetle screen to be required in the same process (column 3) to assemble a set of genes comprising all genes currently known to be required in any insect for the processes analyzed here (column 4). Of the fly gene set (column 1) about two thirds had been tested in the iBeetle screen. Of those, three quarters showed a similar function in our beetle while one quarter appeared to be fly specific (column 2). The subdivisions of columns 1 and 2 are based on Figs. 2 and 3 and Additional file 2: Fig. S1. From the numbers in columns 1-3 we calculated the portions of genes of the combined insect gene set (column 4), which were detected only in *Drosophila* (11%), only in *Tribolium* (37%), or in both (52%). See text for details and discussion of potential systematic biases. B) Respective values for the single processes show that the *Tribolium* screening platform revealed 20–50% novel genes relevant for a process (i.e., which were not detected in *Drosophila*). See Additional file 5: Table S4 for calculations. Given these results neither model system can be used alone as a proxy for insects or protostomes in general and that *Tribolium* is a very useful complementary screening platform

**Fig. 5:**
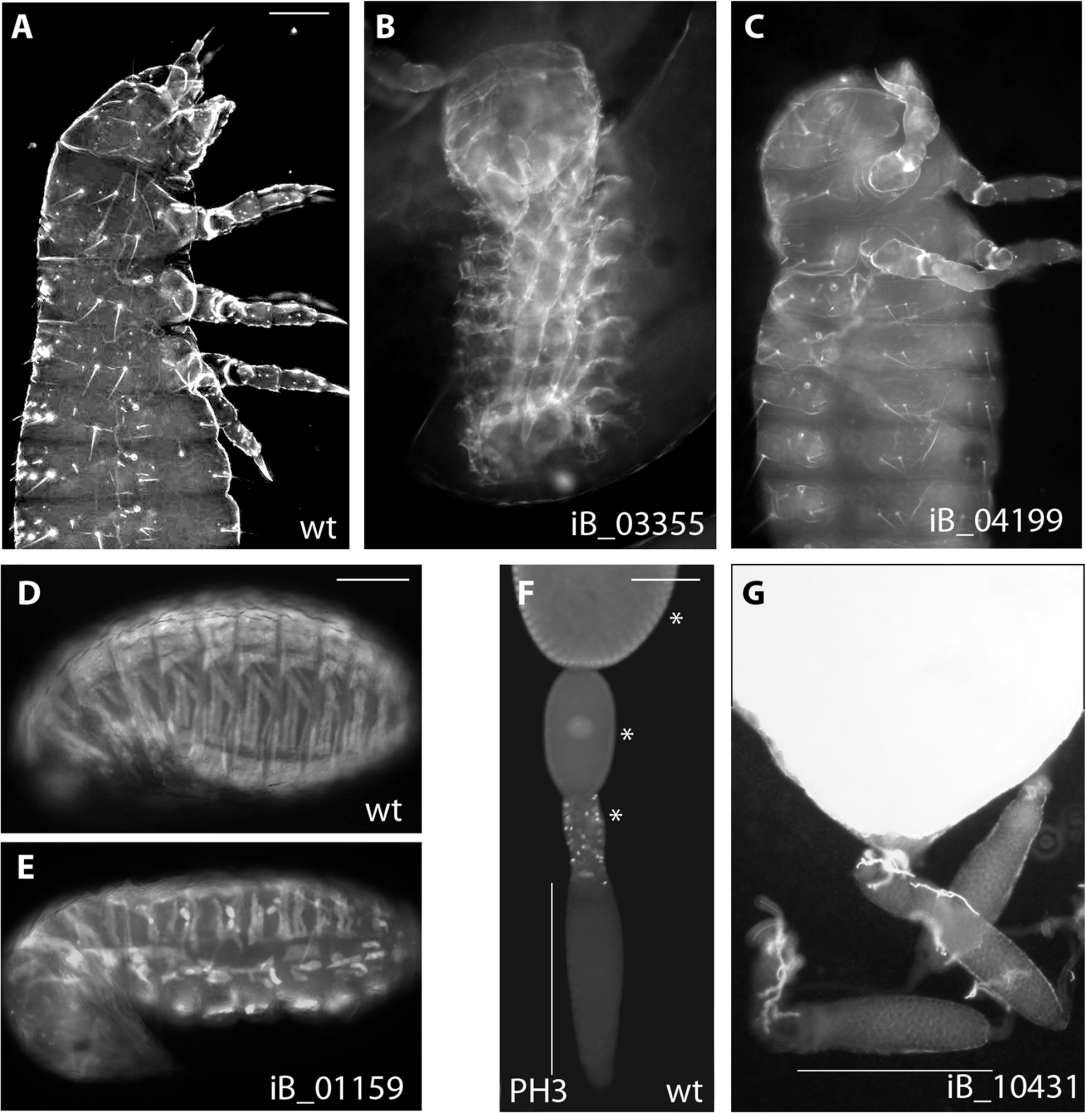
Examples for novel gene functions detected in *Tribolium* but not known from *Drosophila.* **A** Anterior part of a wildtype cuticle with head, thorax, and anterior abdomen. **B** In iB_03355 knock-down embryos, dorso-ventral patterning was disturbed such that the embryo has turned inside out, i.e. the legs and the head are located inside the trunk cuticle instead of outside. **C** In iB_04199 knock-down, anterior epidermal patterning was disrupted to different degrees. In mild phenotypes, just the most anterior part, the labrum, was affected (not shown), intermediate phenotypes lacked head and parts of the thorax (shown) and in strong phenotypes, only cuticle crumbs remained. **D** In the transgenic strain pBA19, the muscles are marked with EGFP. They are visible in vivo as elongated structures with a segmentally repeated pattern. **E** In iB_01159 (*Tc-Unc-76* ) knock-down, the muscles were partially missing or detached such that some muscles adopted a rounded shape. **F** In wildtype ovaries, the nurse cells are located in the tropharium forming an elongated structure (marked by a white line). The first part of the vitellarium is marked by active cell division (marked here by phospho-histone 3 staining, PH3) and along the entire vitellarium, the oocytes increase in size (compare stars). **G** In iB_10431 knock-down ovaries, the tropharia were normal (white line) but no oocytes developed. The white structure is not part of the ovaries. Anterior is to the top in **A**–**E**, the pictures of the phenotypes are modified from iBeetle-Base. Scale bars are 100 μm

Our systematic screening in a complementary model organism has revealed that the identified gene sets show an unexpected degree of divergence (see Fig. 4 for numbers, Fig. 5 for examples). Based on our calculations (see details below) we estimate that only half of the gene functions are detected in both species (52%, column 4 of Fig. 4A) while the remaining gene functions were found either only in *D. melanogaster* (11%, column 4 of Fig. 4A) or only in *T. castaneum* (37%, column 4 of Fig. 4A). We found no strong indication that the gene inventory required for a process would be more conserved for those processes, which seem more conserved morphologically. For instance, dorso-ventral patterning, which we assumed representing an intermediate degree of conservation showed the largest common gene set while the supposedly most conserved process, muscle formation, showed the lowest value. However, we note that we found more *Tribolium*-specific gene functions for the less conserved processes than for muscle development. However, given the uncertainties with these numbers (see the “Discussion” section below) and the fact that morphological conservation of a process is hard to quantify, we hesitate to draw conclusions about the correlation of divergence of a biological process and the involved gene inventory.

While these data were gained with respect to developmental processes only, they strongly indicate that our current knowledge based on screening in one species appears to be much less comprehensive than previously thought. We believe that the different proportions of genes shown to be required for a specific process in only one species (11% vs. 37%) may reflect both, biological and technical differences (see detailed discussion below).

Beyond the fly-beetle comparison, our findings provide a compelling argument that focusing on single model species falls short of comprehensively revealing the genetic basis of biological processes in any clade separated by an evolutionary distance similar or larger than the one separating flies and beetles (i.e., around 370 million years). Further, it shows that *T. castaneum* is an extremely useful screening system for insect biology, able to reveal novel gene functions even in processes that have been studied intensely in *D. melanogaster*.

### Estimating the portions of gene functions revealed in fly versus beetle

In order to make a comprehensive and quantitative comparison, we included in our comparisons all genes that are currently known to be involved in the respective processes from both, beetle and fly. Our beetle data are based on both, our systematic screening of 51% of the *T. castaneum* gene set and on previous candidate gene work. With respect to fly data, we rely on information available on FlyBase and our expert knowledge of the processes under scrutiny. Given these different kinds of sources and approaches, and the fact that we focus on developmental processes, the data are – despite the comprehensive approach - prone to various types of uncertainties. In the following, we first discuss the way we combined the numbers to calculate our estimation. Subsequently, we will discuss some uncertainties and in how far they influence the estimation.

Of the genes known from *D. melanogaster* to be required for the processes investigated here (*n* = 132; see Additional file 5: Table S4), we could compare 66% to *iBeetle* data (column 1 in Fig. 4A; based on Additional file 2: Fig. S1; *n* = 87). Of those genes, 26% (*n* = 23) were not required for that process in *T. castaneum* (column 2 in Fig. 4A; based on Fig. 3). With this statement, we mean that the respective genes did not have any phenotype with respect to the biological process in question. They could have either no phenotype or a phenotype affecting another process. Based on our positive controls, the potential error affecting this statement is less than 7.5% (see Additional file 1: Table S1). For our overall estimation, we extrapolated this share to the total number of genes required for the fly (dotted lines from column 2 to column 4). A number of gene functions detected in the iBeetle screen had not been assigned such functions in *D. melanogaster* before (column 3 in Fig. 4A; based on Fig. 2). When combining these numbers, we aimed at providing a minimum estimation for the divergence of detected gene functions (Column 4 in Fig. 4A). To be conservative, we assumed that all gene functions known from *D. melanogaster* but not yet tested in the iBeetle screen would fall into the class of genes being required for both species (see numbers in green square in Additional file 5: Table S4). Further, we scored each signaling pathway as one case (finding mostly conservation) even if single components of these pathways did not have divergent phenotypes. This conservative assumption leads to the abovementioned minimum estimation of divergence in these gene sets (Column 4 in Fig. 4A; calculation in Additional file 5: Table S4). Of all genes currently known to be required for one of the processes we studied, the portion of genes detected exclusively in the fly (11%; *n* = 23) is much smaller than the one detected only in the beetle (37%; *n* = 76) while the analogous function of half of the genes (52%; *n* = 109) is detected in both species.

With this work, we present the first and a quite extensive dataset to estimate this kind of numbers. Still, some confounding issues need to be considered. The first uncertainty stems from the fact that the beetle data is based on testing about 50% of the genes. In the second part of the screen, we had prioritized genes that were moderately to highly expressed, showed sequence conservation, and had GO annotations. The prioritization apparently was successful as 66% of the gene functions known from *D. melanogaster* had been covered in the iBeetle screen (Fig. 4A), which is much more than the 40% expected for an unbiased selection [17]. Hence, our figures might be biased towards conserved gene function. As a consequence, the overall portion of beetle-specific genes without conserved functions likely is even higher than reflected in Fig. 4A.

Second, we found quite different numbers for the four processes under scrutiny (Fig. 4B). However, even in the process with the lowest portion of genes detected exclusively in *T. castaneum* (muscle development), this portion was 21%, which still indicates a significant degree of unexplored biology.

Third, the *D. melanogaster* numbers could be influenced by false negative data. The data on FlyBase has not been gathered in one or few standardized screens where all data were published—it is mainly based on published results of single gene analyses. However, not all genetic screens have reached saturation and not all genes detected in large-scale screens may have been further analyzed and published. Hence, the number of genes in principle detectable in *D. melanogaster* might actually be larger than the numbers extracted from FlyBase. In the iBeetle screen, in contrast, negative data was systematically documented, such that this type of uncertainty is restricted to technical false negative data, which we found to be around 15% in this first pass screen (Fig. 1). This uncertainty could potentially increase the portion of *D. melanogaster-*specific or conserved genes. Fourth, theoretically, there may be false positive data albeit restricted to the set of genes detected in both species. The reason is that iBeetle was a first pass screen, where we aimed at reducing false negative data with the tradeoff that false positive data are enriched [17]. Although finding similar phenotypes in two different species will not in many cases be false positive, we tried to minimize this error by manually checking the annotations of the respective genes, excluding those that showed a phenotype with low penetrance or in combination with many other defects indicating a non-specific effect. Of note, the issue of false positives is restricted to the genes detected in both species (column 2; based on Fig. 3). It does not apply to those genes detected only in the beetle but not the fly (column 3; based on Fig. 2) because in this case, all phenotypes were confirmed by independent experiments with non-overlapping dsRNA fragments in different genetic backgrounds such that false positive results are excluded. In summary, while there are a number of uncertainties that we could not clarify with available data or methods, most of these uncertainties hint at underestimation rather than overestimation of functional divergence between fly and beetle.

Our work focused on developmental processes with different grades of assumed conservation and different grades of previous knowledge. Morphologically, the muscle pattern and general development appear to be a quite conserved between these insects [31, 49] compared to oogenesis where a number of morphological differences were described [40–42]. Given the background of a strongly derived head morphology of first instar larvae but conserved adult heads and brains, both conservation and divergence were found with respect to the genetic control [6, 36, 50, 51]. Likewise, dorso-ventral patterning is relying on both, conserved and diverged gene regulatory networks [14, 52, 53]. Taken together, our selection appears to cover both conserved and diverged processes such that—at least for the genetic control of development—our data can be generalized with some confidence.

### Technical characteristics contribute to the detection of unequal gene sets

Our numbers reveal that functionally comparable gene sets in two quite closely related model systems are far from identical. A question of obvious biological relevance but not easily resolved is: to which degree do these differences reflect the biologically meaningful divergence of gene functions, or alternatively, simply result from technical problems, i.e., reflect different strengths and weaknesses of the respective screening methods and model systems?

As discussed above, some degree of false negative data may be expected in both model systems. In the case of the iBeetle screen, this will be restricted to technical false negative data. In the *D. melanogaster* field, there may be additional false negative data due to the lack of saturation of screens and/or lack of reporting of genes that were not studied in detail. However, given the extent and comprehensiveness of work in the *D. melanogaster* field, we feel that this might not be of high relevance. As to the different strengths of screening procedures, it is certainly true that the way screens are performed influences what sets of genes can be detected. For instance, our parental RNAi approach knocked down both, maternal and zygotic contributions while some classic *D. melanogaster* screens affected only the zygotic contribution. Hence, genes where maternal contribution rescues the embryonic phenotype are easily missed in the fly but not the beetle. For instance, parental RNAi knocking down components of the aPKC complex leads to severe early disruption of embryogenesis in *T. castaneum* while in respective *D. melanogaster* mutants almost no defects are seen on the cuticle level (A. Wodarz, unpublished observation). Conversely, our RNAi screen depended on the accuracy of gene annotations and our approach of screening for several processes in parallel may have reduced detection sensitivity. One striking example of the different strengths of screening designs is provided by wing blister phenotypes. In the first part of the *iBeetle* screen, we detected 34 genes showing wing blister phenotypes where 14 did not have related GO term annotation at FlyBase and 5 did not have any GO annotation at all. Seven of these genes were subsequently tested by RNAi lines in *D. melanogaster* where four of them indeed showed a related phenotype. Likewise, some wing blister genes from *D. melanogaster* were not annotated in the iBeetle screen. When we checked more specifically, this was often due to the lethality of the animal before the formation of wings [17]. When we varied the timing of injection, two of those knock-downs elicited wing blister phenotypes also in *T. castaneum* [17]. These data show that details of the screening procedure influence the subset of genes that are detected.

### Evolutionary divergence of gene function and derived *Drosophila* biology may be larger than appreciated

Most relevant for the field of functional genetics is our conclusion that the degree of divergence of gene functions among holometabolous insects is larger than previously assumed. Therefore, some genes are detected only in one species because the gene’s function is not required for that process in the other. This finding should of course influence our thinking about using any insect as model for human development and diseases such as muscle fomation and congenital heart defect.

Indeed, there is evidence supporting the notion of an unexpected degree of divergence with respect to muscle development. Based on the *iBeetle* screen, a number of muscle genes identified in the *iBeetle* screen were more closely investigated in *D. melanogaster* [31, 49]. Despite quite some efforts, the negative data for fly orthologs appeared to be true negative. For example, null mutations of one of the genes found in our beetle, *nostrin*, did not elicit a phenotype in *D. melanogaster* unless combined with a mutation of a related F-bar protein *Cip4.* Likewise, *Rbm24* displays strong RNAi and mutant phenotypes in *T. castaneum* and vertebrates, respectively, but *D. melanogaster* is lacking an *Rbm24* ortholog, and functional compensation by paralogs was suggested to occur during *D. melanogaster* muscle development. Other genes including *kahuli* and *unc-76* are expressed in the *D. melanogaster* mesoderm but only showed very subtle somatic muscle phenotypes, if any, in Mef2-GAL4 driven RNAi experiments or with CRISPR/Cas9 induced mutations, respectively (see Materials & Methods). By contrast, their beetle counterparts had strong and penetrant phenotypes in single knock-downs (e.g. see *Tc-unc-76* in Fig. 5E) [31, 49, 54]. These data suggest that the function of genes or their relative contribution to this biological process has changed significantly. They also indicate that the single gene view may be limited. Phenotypes depend on networks of interacting genes and this may allow for changes and replacements of individual components while the overall network structure is maintained. There are more striking examples of gene function changes. The gene *germ cell-less* was detected in the iBeetle screen to govern anterior-posterior axis formation in the beetle while in *D. melanogaster* it is required for the formation of the posterior germ-cells [51]. Also, the *D. melanogaster* textbook example of a developmental morphogen *bicoid* does not even exist in *T. castaneum* [5] and yet other genes were found to act as anterior determinants in other flies [9, 10]. Along the same lines, the genes *forkhead* and *buttonhead* do not appear to be required for anterior patterning in *T. castaneum* but are essential in flies [12, 39, 55, 56].

These findings with respect to specific genes add to a number of observations arguing for a comparatively high degree of divergence due to the overall highly derived nature of fly biology. The number of genes is much smaller in *D. melanogaster* (appr. 14,000) compared to *T.castaneum* (appr. 16,500). Further, a number of developmental processes are represented in a more insect-typical way in *T. castaneum* like for instance segmentation [57], head [50] and leg development, brain development [58], extraembryonic tissue movements [59], and mode of metamorphosis [60]. In most cases, the situation in the fly is simplified and appears to be streamlined for faster development. We think that these biological differences might be the basis for divergence in gene function, which we just started to uncover. In the absence of similar large-scale comparisons in other species, it remains open, whether an insect-typical gene set even exists or whether one would rather have to emphasize a constant change of gene function, such that any ancestral gene set simply “melts” away with evolutionary time.

## Conclusion

We found that the gene sets detected for the same processes in flies and beetles differ much more than expected—only about 50% of the genes were detected in both species. Given the large divergence of gene sets found in different screening systems and the documented cases of biological divergence of gene function, we propose that a more systematic investigation on the divergence of gene function is needed. Further, the hypothesis independent screening now possible in *T. castaneum* may be very helpful in that endeavor.

## Methods

### Screen

We followed the tested and published procedures apart from some minor changes detailed below (please find an extensive description of the procedure in Schmitt-Engel et al. [17]). In particular, we used the same strains, injection procedures, and incubation temperatures and incubation times. We injected 10 pupae per gene leading to the collection of > 50 offspring L1 cuticles, which were analyzed. Phenotypes were annotated and their penetrance documented. For subsequent work, we only considered phenotypes, which showed a penetrance of > 50%. dsRNAs (1 μg/μl) were produced by Eupheria Biotech Dresden, Germany. Different from the published procedure, the stink gland analysis was performed 21 days after pupal injection (in the first screening phase, this analysis had been performed after larval injection).

### Controls of the screen

To assess the sensitivity and reliability of the screen, and to test the accuracy of each screener, we included approximately 5% positive controls randomly chosen from a set of 35 different genes. By and large, we used the same positive controls as in the first screening phase (see Table Table_S1_controls). However, *Tc-zen-1* was excluded since the phenotypes were much weaker than in the previous screen, probably due to the degradation of the dsRNA. We added new positive controls to score for muscle and stink-gland phenotypes, which we took from novel genes detected in the first screening phase. New controls for muscle phenotypes: iB_06061, iB_05796, iB_03227, iB_01705; for stink gland and ovary phenotypes: iB_02517; for head defects: iB_05442 (that gene was not scored for its stink gland phenotype because it turned out to be too mild to be identified reliably in high throughput). In 143 cases (80.8%, *n* = 177), the phenotypes of positive controls were fully recognized (for comparison: in the first screening phase the respective numbers were: 90%, *n* = 201). In 14 cases (7.9%; phase 1: 4%) the phenotype was partially recognized. This category includes complex phenotypes where half (one of two aspects: *knirps*, *piwi*, *SCR*, *cta*, *cnc*, *iB_01705*, *iB_05442*) or two of three aspects (*aristaless*) of all phenotypic aspects were correctly identified. 13 phenotypes were missed completely (7.3%, phase 1: 4% ). *Tc-metoprene tolerant (Tc-met)* was missed most frequently, probably due to the fact that the embryonic leg phenotype was very subtle and in addition, the penetrance of the phenotype appeared to be lower than in the first screen (penetrance: less than 30%). Seven positive controls (4%, phase 1: 1%) could not be analyzed due to prior technical lethality, i.e. the premature death of the injected pupae prevented the detection of the phenotype. In three cases wrong aspects were annotated (false positive: 1.7%). Depending on the other annotations these positive controls were valued as partially recognized (SCR) or missed (met, CTA). Find more details in Table Table_S1_controls.

Negative controls (buffer injections) were mainly annotated correctly (no phenotype in 92.9%; phase 1: 96%) and just in 7 cases led to false positive annotations (7.1%; phase 1: 2%) (Table Table_S1_controls; sheet 2).

### Re-screen

Re-screening of selected iBeetle candidates was performed in order to probe for off-target and strain-specific effects. For that purpose, two independent dsRNA fragments (original iB-fragments and one non-overlapping fragment, both at concentration 1 μg/μl) targeting the same gene were injected separately into a different genetic background (*San Bernardino*, *SB* strain), except for the muscle project where it is required to use the pBA19 strain with EGFP marked muscles. The rest of the injection procedures and analyses were performed like in the screen. Note that with this approach, we cannot exclude that phenotypes observed in one tissue are elicited by knock-down in another tissue (e.g., hormone-induced morphogenesis may fail due to knock-down of hormone production in a gland).

### Fly gene sets

Lists of genes involved in those processes were established by our experts of the respective processes. This was supported by individual FlyBase searches for respective GO terms of the category “biological process”. For the analyses in Figs. 2 and 3, we only considered gene functions, which were based on experimental data as documented at FlyBase in the year 2017 (Dmel Release 6.18) with updates in single case in 2020 (Dmel Release 6.32).

### Phylogenetic analysis

The *Tribolium* protein sequences from gene set OGS3_proteins.fasta.gz (including changes from 2016/02/15, available from [27]) were used to retrieve the most similar proteins of *T. castaneum*, *D. melanogaster*, and *M. musculus* using only one isoform. Multiple alignments were done with the ClustalOmega plugin as implemented in the Geneious 10.1.3 software (Biomatters, Auckland, New Zealand) using standard settings. Alignments were trimmed to remove poorly aligned sequence stretches. Phylogenetic trees were calculated using the FastTree 2.1.5 plugin implemented in Geneious.

### Generation of Unc-76 mutations via CRISPR/Cas9

In order to generate the *Unc-76* mutations, we essentially followed the procedure described by Basset et al., 2013 [61]—please find an extensive description there. For making the template for the guide RNAs, the *Unc-76* target sequence GGTTCAACGATCTGACCAGTG was inserted between the T7 promoter and the gRNA core sequence in the forward primer, gRNA_F. After annealing gRNA_F with SGRNAR, the template was PCR amplified with Q5 polymerase (NEB). Guide RNAs were transcribed with Ampliscribe T7 Flash (epicenter), isolated with the MEGAclear kit (Ambion), and injected together with Cas9 mRNA into *w*[1118] *sn*[3] *P{ry+t7.2=neoFRT}19A* embryos. Single lines established from the offspring were tested as heterozygotes over the balancer *FM7c*. We used a T7 endonuclease assay for detecting sequence alterations near the target site as described in [62]. Our lethal *Unc-76*[CR007] allele carries a 16 nucleotide deletion near the target site in the sequence ..TAT CCA CAC ACc aac ggt ttg gga tcc GGA TCC GGA TCC.. of the second exon (X: 2091152... 2091167, r6.32; lower case letters represent the deleted DNA) that creates a frameshift in the ORF of all known isoforms (i.e., the frameshift occurs after T246 in Unc-76 RA to -C and after T61 in Unc-76 RD).

## Supplementary Information

**Additional file 1: Table S1.** Positive and negative controls of the screen.

**Additional file 2: Figure S1.** Diagram displaying the portion of genes known to be required for the processes in *Drosophila,* which were tested in Tribolium. **Figures S2 and S3.** Phylogenetic trees supporting our claim of absence of an ortholog in *Drosophila*.

**Additional file 3: Table S2.** Genes with phenotype in *Tribolium* but not *Drosophila.*

**Additional file 4: Table S3.** Genes with phenotype in *Drosophila* compared to *Tribolium* data.

**Additional file 5: Table S4.** Combined analysis leading to the numbers given in Figure 4.

## Data Availability

The datasets generated and/or analyzed during the current study are available from the iBeetle-Base [25, 26] repository [27]. The dsRNA fragments used to knock down the genes are commercially available from Eupheria Biotech, their sequences are documented in the iBeetle-Base.
