# Supplementary material for: Screens in fly and beetle reveal vastly divergent gene sets required for developmental processes
Source: BMC Biol. 2022 Feb 8;20:38. doi: 10.1186/s12915-022-01231-4 (PMC8827203; doi:10.1186/s12915-022-01231-4)

Figure S1: Genes known from Drosophila tested in the iBeetle screen

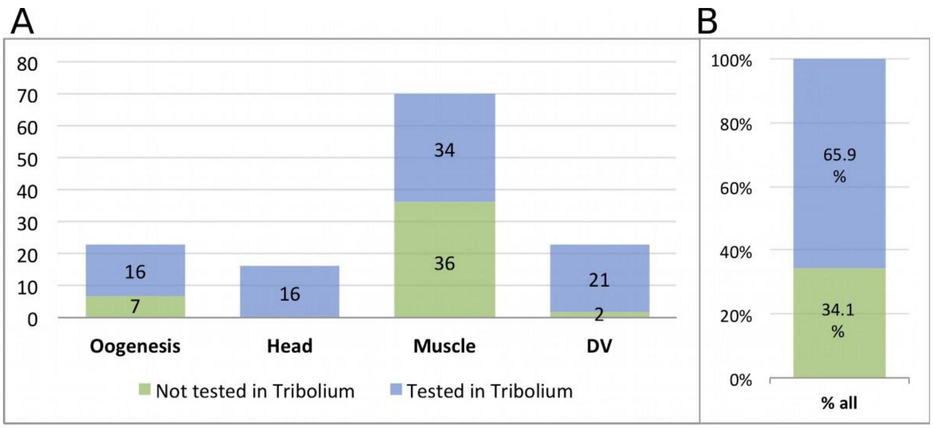

Figure S2: Phylogenetic_Tree_TC001720

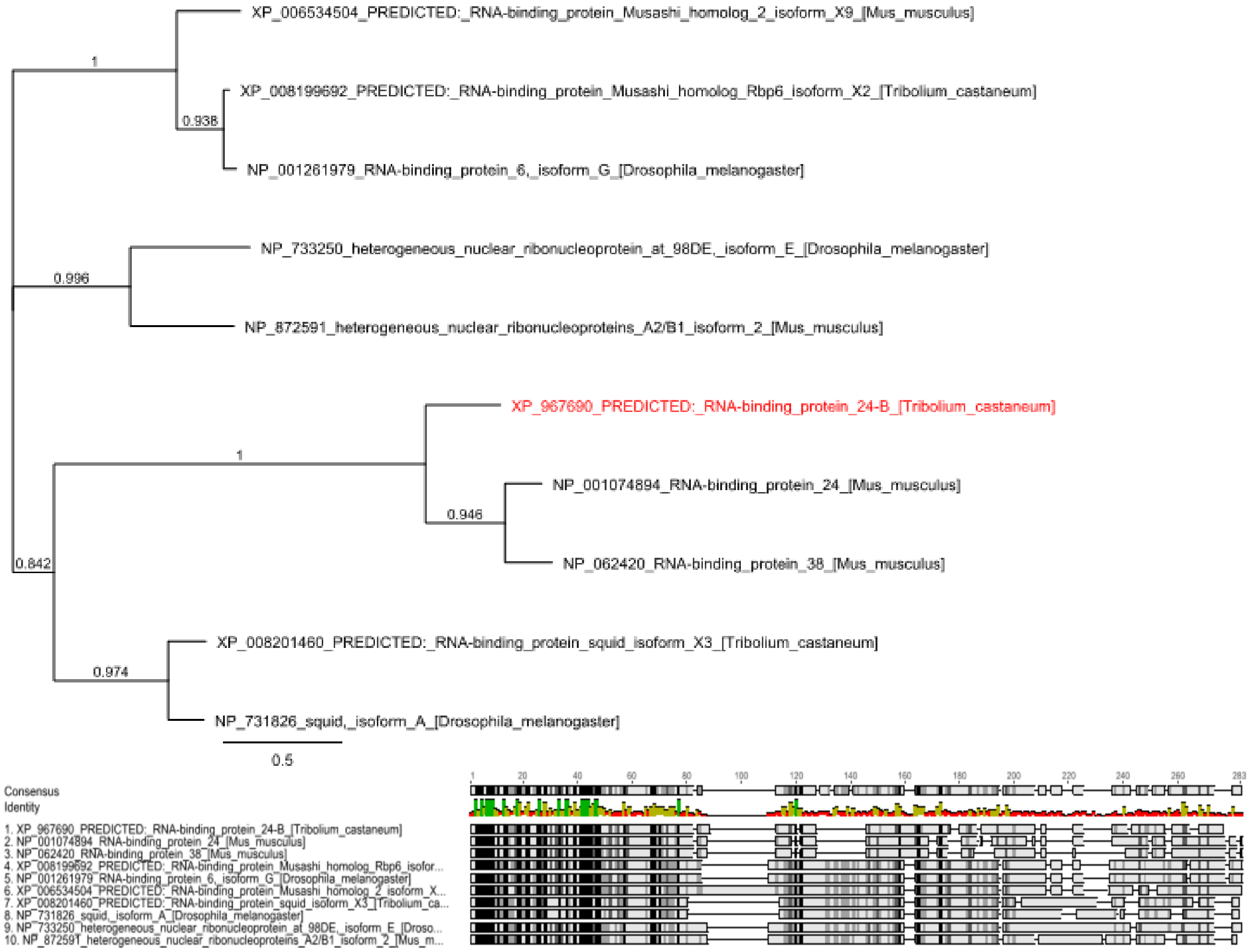

Figure S3: Phylogenetic_Tree_TC002909

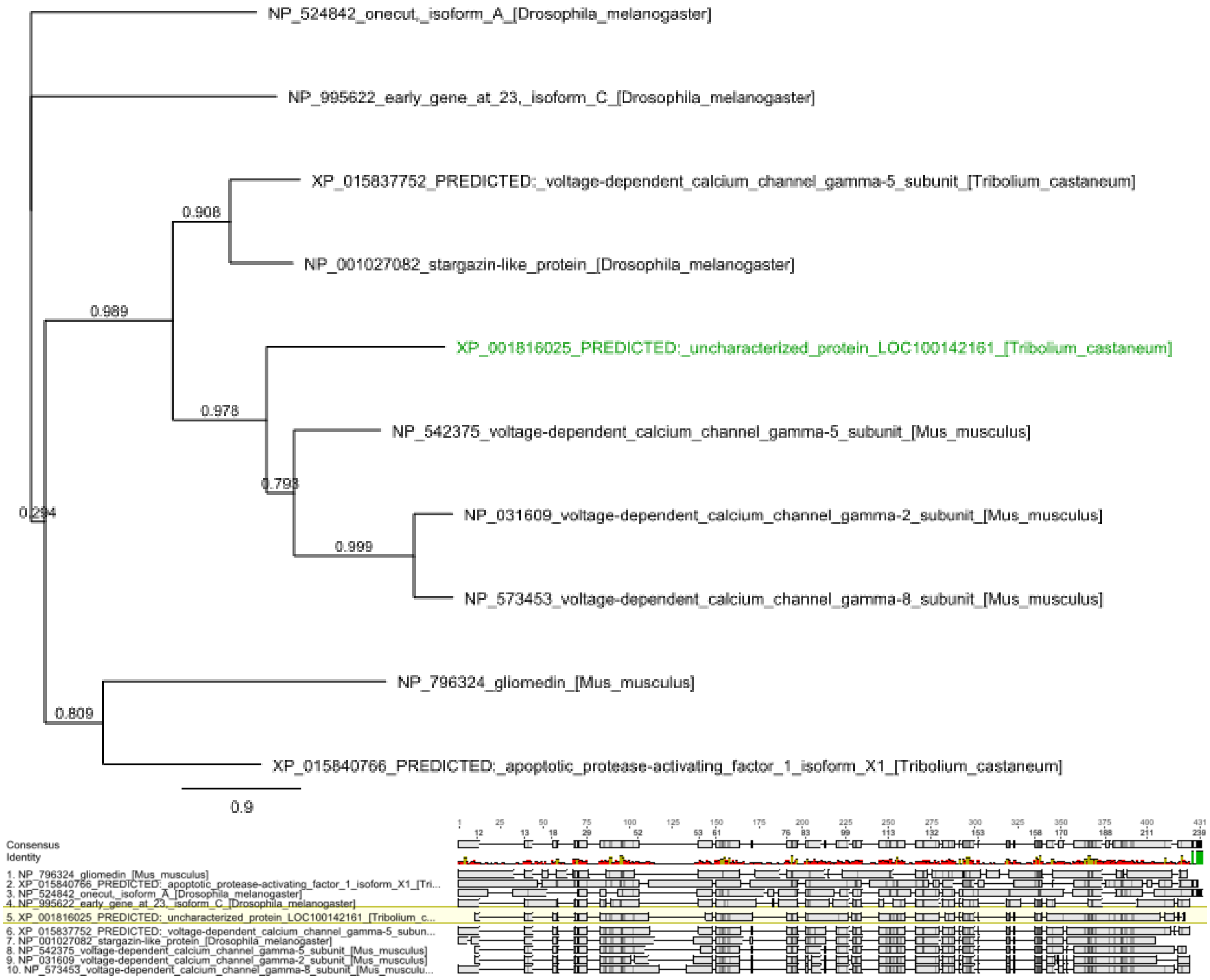

Supplement: Supplementary file 2 — Additional file 2: Figure S1. Diagram displaying the portion of genes known to be required for the processes in Drosophila, which were tested in Tribolium. Figures S2 and S3. Phylogenetic trees supporting our claim of absence of an ortholog in Drosophila. [file 12915_2022_1231_MOESM2_ESM.pdf]
